# Berberine Induces Mitophagy through Adenosine Monophosphate-Activated Protein Kinase and Ameliorates Mitochondrial Dysfunction in PINK1 Knockout Mouse Embryonic Fibroblasts

**DOI:** 10.3390/ijms25010219

**Published:** 2023-12-22

**Authors:** Jee-Hyun Um, Kang-Min Lee, Young-Yeon Kim, Da-Ye Lee, Eunmi Kim, Dong-Hyun Kim, Jeanho Yun

**Affiliations:** 1 Department of Biochemistry, College of Medicine, Dong-A University, Busan 49201, Republic of Korea; umj1127@dau.ac.kr (J.-H.U.); kmlee8348@dau.ac.kr (K.-M.L.); kyy8191@dau.ac.kr (Y.-Y.K.);; 2 Department of Translational Biomedical Sciences, Graduate School of Dong-A University, Busan 49201, Republic of Korea; 3 Department of Pharmacology, School of Medicine, Konkuk University, Seoul 05029, Republic of Korea; mose79@kku.ac.kr; 4 Department of Advanced Translational Medicine, School of Medicine, Konkuk University, Seoul 05029, Republic of Korea

**Keywords:** berberine, mitophagy, mitochondrial dysfunction, Parkinson’s disease

## Abstract

Mitophagy stimulation has been shown to have a therapeutic effect on various neurodegenerative diseases. However, nontoxic mitophagy inducers are still very limited. In this study, we found that the natural alkaloid berberine exhibited mitophagy stimulation activity in various human cells. Berberine did not interfere with mitochondrial function, unlike the well-known mitophagy inducer carbonyl cyanide m-chlorophenyl hydrazone (CCCP), and subsequently induced mitochondrial biogenesis. Berberine treatment induced the activation of adenosine monophosphate-activated protein kinase (AMPK), and the AMPK inhibitor compound C abolished berberine-induced mitophagy, suggesting that AMPK activation is essential for berberine-induced mitophagy. Notably, berberine treatment reversed mitochondrial dysfunction in PINK1 knockout mouse embryonic fibroblasts. Our results suggest that berberine is a mitophagy-specific inducer and can be used as a therapeutic treatment for neurodegenerative diseases, including Parkinson’s disease, and that natural alkaloids are potential sources of mitophagy inducers.

## 1. Introduction

Mitophagy, the selective degradation of damaged or old mitochondria, has emerged as a critical cellular process with far-reaching implications for maintaining cellular homeostasis and addressing various human diseases, including neurodegenerative diseases [1,2]. The controlled removal of impaired mitochondria through mitophagy serves as a quality control mechanism, preventing the accumulation of dysfunctional organelles and safeguarding cellular integrity [3,4]. Dysregulation of mitophagy has been implicated in various neurodegenerative disorders, including Parkinson’s, Alzheimer’s, and Huntington’s diseases, highlighting its central role in mitigating mitochondrial dysfunction [5]. Mitochondrial dysfunction, characterized by impaired membrane potential, elevated reactive oxygen species (ROS) production, and compromised respiratory capacity, is a hallmark of neurodegenerative diseases [6,7]. The induction of mitophagy presents a promising avenue for therapeutic interventions, as it facilitates the selective removal of damaged mitochondria and promotes the maintenance of a healthy mitochondrial pool within cells [5]. Consequently, this process contributes to the preservation of cellular energy production, a reduction in oxidative stress, and overall improvement in cellular function [8]. Despite the recognized therapeutic potential of mitophagy, the search for nontoxic mitophagy inducers remains an ongoing challenge [1,9]. The limited repertoire of such compounds underscores the importance of identifying novel agents that can specifically modulate mitophagy without adverse effects on cellular function [10].

Berberine is a natural alkaloid with a rich history of traditional use in various medicinal practices, particularly in Ayurvedic and Chinese medicine [11]. Derived from the roots and bark of certain plants, such as *Berberis*, *Coptis*, and *Phellodendron*, berberine has garnered substantial attention in contemporary research due to its diverse pharmacological properties [12]. Studies have explored the potential of berberine in addressing a range of health conditions, revealing anti-inflammatory, antidiabetic, and cardiovascular benefits [13,14]. While previous studies have implicated berberine’s involvement in mitochondrial function and mitophagy regulation [15], a conclusive understanding of its precise role has remained elusive.

In this study, we explored the mitophagy-stimulating function of berberine. Through systematic examination, we demonstrated that berberine possesses the ability to induce mitophagy across various human cells without compromising mitochondrial function or cellular viability. Intriguingly, we also revealed that berberine-induced mitophagy is mediated by adenosine monophosphate (AMP)-activated protein kinase (AMPK), a pivotal regulator of mitophagy induction. Moreover, we further showed that berberine efficiently reversed mitochondrial dysfunction in PTEN-induced kinase 1 (PINK1) knockout (PINK1 KO) mouse embryonic fibroblasts (MEFs). These results provide direct evidence that berberine promotes mitophagy with low toxicity for the first time and that berberine has the potential to effectively improve mitochondrial dysfunction.

## 2. Results

### 2.1. Berberine Induces Mitophagy in Human Cells

**Figure 1:**
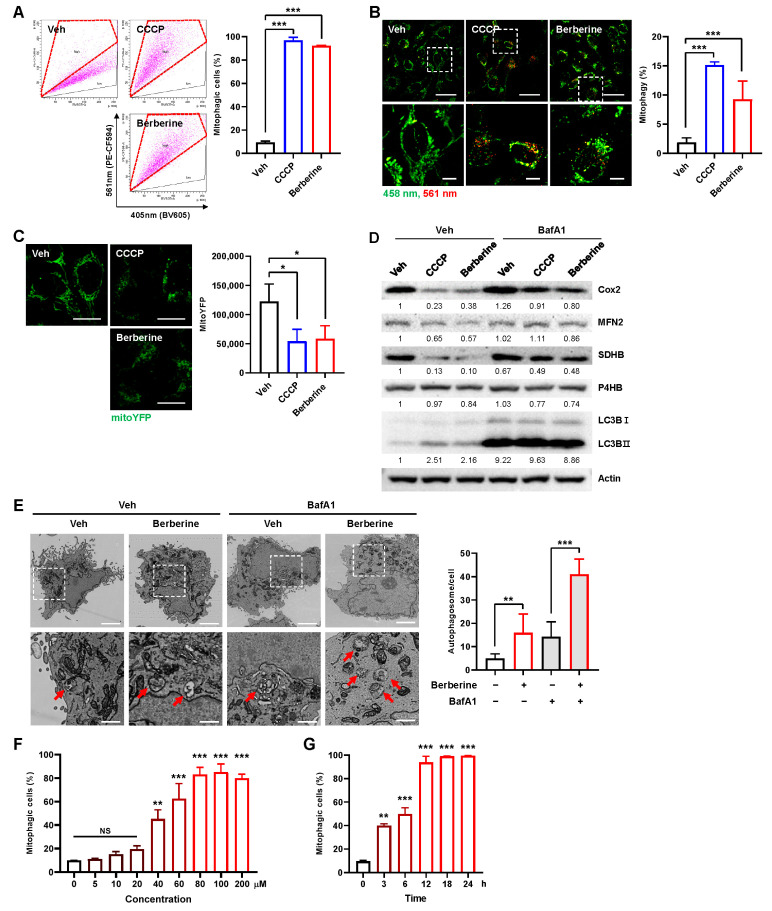
Verification of mitophagy induction by berberine. (**A**–**D**) BEAS-2B cells expressing mt-Keima (**A**,**B**), BEAS-2B cells expressing mitoYFP (**C**) or BEAS-2B cells (**D**) were treated with berberine (80 μM) or CCCP (10 μM) for 24 h. Veh: vehicle. Mitophagy levels were analyzed by flow cytometry (**A**). The results from three biological replicates are shown as the mean ± SD. Mitophagy levels were analyzed by confocal microscopy (**B**). The results from three biological replicates are shown as the mean ± SD. Scale bar: 50 μm (top). The boxed regions are enlarged in the bottom panel. Scale bar: 20 μm (bottom). The fluorescence intensity of mitoYFP was analyzed by confocal microscopy (**C**). Quantified fluorescence intensities from three biological replicates with several images per biological repeat are shown on the right as the mean ± SD. Scale bar: 10 μm. Cell lysates were subjected to Western blot analysis using the indicated antibodies (**D**). Numbers below the corresponding blot represent densitometry values normalized to actin. (**E**) BEAS-2B cells were treated with berberine (80 μM) with or without bafilomycin A1 (BafA1; 100 nM) for 12 h and analyzed by transmission electron microscopy. Scale bars: 3 μm (top). Arrows indicate autophagosomes containing mitochondria. Scale bar: 1 μm (bottom). The number of autophagosomes per cell is shown on the right as the mean ± SD (n ≧ 10 per sample). (**F**,**G**) BEAS-2B cells expressing mt-Keima were treated with berberine at the indicated concentration for 24 h (**F**) or treated with berberine (80 μM) for the indicated periods (**G**), and mitophagy levels were analyzed by flow cytometry. The results from repeated experiments are shown as the mean ± SD. Significance was determined by one-way ANOVA with Šidák’s multiple-comparison test. * *p* < 0.05; ** *p* < 0.01; *** *p* < 0.001. NS, not significant.

To investigate the mitophagy induction activity of berberine, we measured mitophagy activity employing a previously established FACS-based mitophagy assay using the pH-dependent fluorescent reporter protein mt-Keima [16,17]. Mitophagy analysis after treating BEAS-2B human nontumorigenic lung epithelial cells with berberine (80 μM) revealed that berberine treatment led to a marked increase in the proportion of cells with a high 405 nm/561 nm ratio, similar to a well-known mitophagy inducer, carbonyl cyanide m-chlorophenyl hydrazone (CCCP), indicating active mitophagy (mitophagic cells (%)) (Figure 1A). Confocal microscopy analysis further revealed a noticeable increase in the red puncta of mt-Keima, indicative of heightened mitophagy [16,18] (Figure 1B). The quantification of mitophagy using a previously established method [16,18] indicated that mitophagy levels were increased approximately 4.9-fold in BEAS-2B cells after berberine treatment (Figure 1B). Furthermore, the fluorescence intensity of mitochondrial YFP (mitoYFP) declined to less than 50% following 24 h of berberine treatment, similar to CCCP (Figure 1C). Treatment with berberine also resulted in a reduction in mitochondrial proteins, including MFN2, SDHB, and Cox2, while endoplasmic reticulum (ER) protein P4HB was not changed (Figure 1D). Berberine treatment also increased the level of the LC3B-II isoform, a typical marker of mitophagy induction. Cotreatment with berberine and the lysosome inhibitor bafilomycin A (BafA1) further increased the amount of the LC3B-II form and inhibited the decrease in mitochondrial proteins (Figure 1D), indicating that berberine induces mitophagy flux.

Moreover, electron microscopy analysis revealed a significant increase in the number of autophagosomes containing mitochondria, observed both in the presence and absence of the lysosome inhibitor bafilomycin A (BafA1) (Figure 1E). The results of a dose-dependent analysis suggested a significant induction of mitophagy by berberine starting at a concentration of 40 μM, peaking at 80 μM (Figure 1F). Additionally, berberine induced mitophagy in a time-dependent manner (Figure 1G). These results suggest that berberine treatment induces mitophagy activity in BEAS-2B cells.

**Figure 2:**
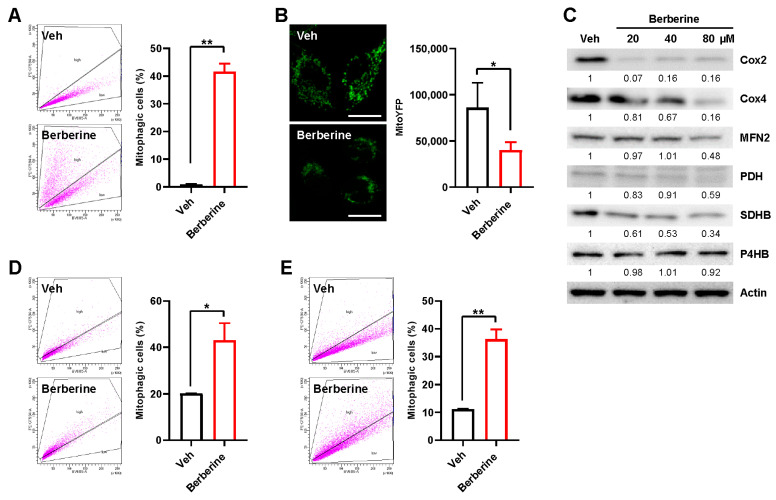
Mitophagy induction by berberine in various human cells. (**A**–**C**) HeLa–Parkin cells expressing mt-Keima (**A**), HeLa–Parkin cells expressing mitoYFP (**B**), or HeLa–Parkin cells (**D**) were treated with berberine (80 μM) for 24 h. Mitophagy levels were analyzed by flow cytometry (**A**). The results from repeated experiments are shown as the mean ± SD. Veh: vehicle. The fluorescence intensity of mitoYFP was analyzed by confocal microscopy (**B**). Quantified fluorescence intensities from three biological replicates with several images per biological repeat are shown on the right as the mean ± SD. Scale bar: 20 μm. Cell lysates were subjected to Western blot analysis using the indicated antibodies (**C**). Numbers below the corresponding blot represent densitometry values normalized to actin. (**D**,**E**) SH-SY5Y cells expressing mt-Keima (**D**) or A549 cells expressing mt-Keima (**E**) were treated with berberine (80 μM) for 24 h. Mitophagy levels were analyzed by flow cytometry. The results from repeated experiments are shown as the mean ± SD. Mitophagy levels were analyzed by confocal microscopy. Significance was determined by Student’s *t* test. * *p* < 0.05; ** *p* < 0.01.

To further validate berberine-induced mitophagy, we assessed mitophagy induction in various human cell lines after berberine treatment. In HeLa cells expressing Parkin, we observed an increase in mitophagic cells, a decrease in mitoYFP, and a reduction in mitochondrial proteins following berberine treatment (Figure 2A–C). Moreover, an increase in mitophagic cells was also noted in SH-SY5Y human neuroblastoma cells and A549 human lung cancer cells after berberine treatment (Figure 2D,E). These results suggest that berberine induces mitophagy in various human cells.

### 2.2. Berberine Does Not Interfere with Mitochondrial Function

**Figure 3:**
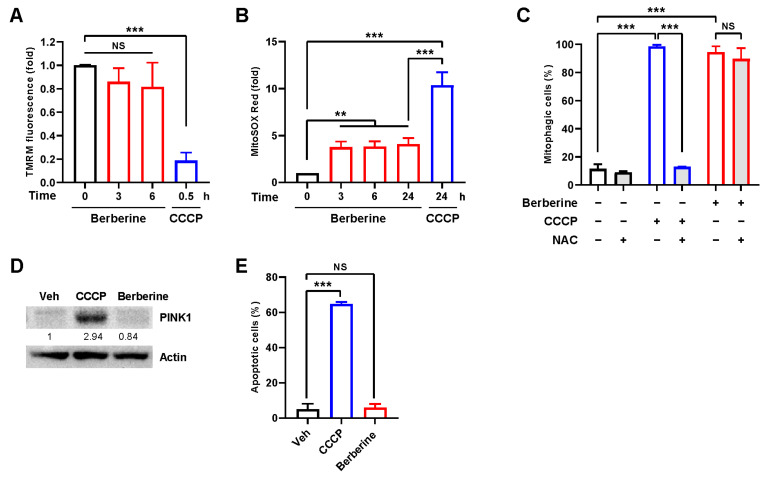
Effect of berberine on mitochondrial function and cell viability. (**A**,**B**) BEAS-2B cells were treated with CCCP (10 μM) or berberine (80 μM) for the indicated periods. The mitochondrial membrane potential was assessed by TMRM staining (**A**), and mitochondrial ROS levels were determined by MitoSOX Red staining (**B**). The results from three biological replicates are shown as the mean ± SD. (**C**) BEAS-2B cells expressing mt-Keima were treated with CCCP (10 μM) or berberine (80 μM) alone or cotreated with N-acetylcysteine (NAC) (2 mM) for 24 h, and mitophagy levels were analyzed by flow cytometry. (**D**) BEAS-2B cells were treated with CCCP (10 μM) or berberine (80 μM) for 24 h. Cell lysates were subjected to Western blot analysis using the indicated antibodies. Numbers below the corresponding blot represent densitometry values normalized to actin. (**E**) BEAS-2B cells were treated with CCCP (10 μM) or berberine (80 μM) for 24 h, and apoptotic cells were analyzed 3 days later by flow cytometry after Annexin V-FITC/PI staining. The results from three biological replicates are shown as the mean ± SD. Significance was determined by one-way ANOVA (**A**,**B**,**E**) or two-way ANOVA (**C**) with Šidák’s multiple-comparison test. ** *p* < 0.01; *** *p* < 0.001. NS, not significant.

Widely used mitophagy inducers such as CCCP and carbonyl cyanide *p*-trifluoromethoxyphenylhydrazone (FCCP) trigger mitophagy by interfering with various mechanisms of mitochondrial function, including the disruption of mitochondrial membrane potential and an increase in mitochondrial reactive oxygen species (ROS) [19]. To investigate the impact of berberine on mitochondrial function, we assessed mitochondrial membrane potential using tetramethylrhodamine methyl ester (TMRM) staining during berberine treatment (80 μM). Interestingly, the mitochondrial membrane potential did not significantly decrease until 6 h of treatment (Figure 3A). A moderate increase in mitochondrial ROS levels was observed upon berberine treatment (Figure 3B). However, berberine-induced mitophagy remained unaffected by cotreatment with the potent antioxidant N-acetylcysteine (NAC) (Figure 3C), suggesting that mitophagy induction upon berberine treatment is not dependent on mitochondrial ROS. In addition, an increase of PINK1 was also not observed upon berberine treatment unlike CCCP (Figure 3D). Moreover, berberine treatment did not induce cell death, while CCCP induced significant cell death (Figure 3E). These results collectively indicate that berberine, in contrast to compounds such as CCCP, does not interfere with mitochondrial function for the induction of mitophagy.

### 2.3. Berberine Specifically Induces Mitophagy through AMPK

**Figure 4:**
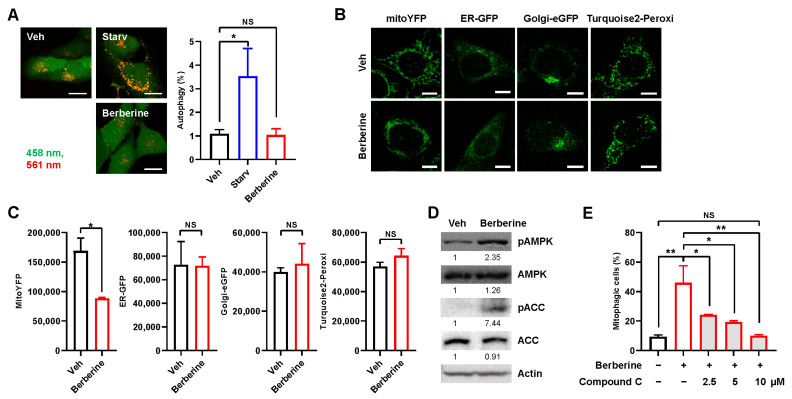
Analysis of mitophagy-specific induction and AMPK-dependency. (**A**) BEAS-2B cells expressing Keima were starved for 3 h (Starv) or treated with berberine (80 μM) for 24 h, and autophagy levels were analyzed by confocal microscopy. Scale bar: 20 μm. (**B**) HeLa–Parking cells expressing mitoYFP, ER-GFP, Golgi-eGFP, or Turquoise2-Peroxi were treated with berberine (80 μM) for 24 h, and the fluorescence intensities were analyzed by confocal microscopy. Scale bar: 10 μm. (**C**) Quantified fluorescence intensities from repeated experiments with several images per biological repeat are shown as the mean ± SD. (**D**) BEAS-2B cells were treated with berberine (80 μM) for 24 h and cell lysates were subjected to Western blot analysis using the indicated antibodies. Numbers below the corresponding blot represent densitometry values normalized to actin. (**E**) BEAS-2B cells expressing Keima were treated with berberine (80 μM) together with compound C at the indicated concentration for 3 h, and mitophagy levels were analyzed by flow cytometry. The results from repeated experiments are shown as the mean ± SD. Significance was determined by one-way ANOVA (**A**,**E**) with Šidák’s multiple-comparison test or by Student’s *t* test (**C**). * *p* < 0.05; ** *p* < 0.01. NS, not significant.

We proceeded to investigate whether berberine specifically induces mitophagy. While mt-Keima can be used to precisely measure mitophagy activity, macroautophagy levels can be assessed using untargeted Keima protein [20,21]. Starvation, a representative inducer of macroautophagy, markedly increased cytosolic Keima puncta, but berberine treatment did not induce Keima puncta (Figure 4A), suggesting that berberine does not induce macroautophagy. Moreover, the analysis of fluorescent markers for different subcellular organelles revealed that only the signal for the mitochondrial marker (mitoYFP) decreased; signals for other organelle markers, such as those for the ER (ER-GFP), Golgi (Golgi-eGFP), and peroxisomes (Turquoise2-Peroxi), were unaffected after berberine treatment (Figure 4B,C). These results further support the hypothesis that berberine specifically induces mitophagy.

Notably, we observed an increase in the phosphorylation of AMPK at Thr172, a marker of AMPK activation, after berberine treatment (Figure 4D). We also found that the phosphorylation of ACC at Ser79, which is a well-known marker for AMPK activation, increased upon berberine treatment (Figure 4D). To investigate whether AMPK activation is essential for the induction of mitophagy by berberine, we examined the impact of the potent AMPK inhibitor compound C [22]. Interestingly, compound C inhibited berberine-induced mitophagy in a dose-dependent manner (Figure 4E). Treatment with 10 μM compound C completely suppressed the induction of mitophagy by berberine, suggesting that AMPK may mediate the induction of mitophagy upon berberine treatment.

### 2.4. Berberine Simultaneously Induces Mitochondrial Biogenesis

**Figure 5:**
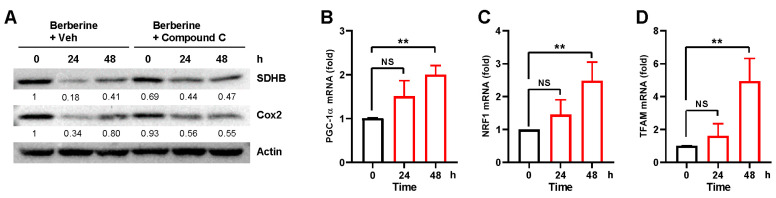
Analysis of mitochondrial biogenesis after berberine treatment. (**A**) BEAS-2B cells were treated with berberine (80 μM) together with vehicle (Veh) or compound C (2.5 μM) for 24 h, and cell lysates were subjected to Western blot analysis using the indicated antibodies. Numbers below the corresponding blot represent densitometry values normalized to actin. (**B**–**D**) BEAS-2B cells were treated with berberine (80 μM) for 24 h. Cells were harvested at the indicated time points, and the mRNA levels of PGC-1α (**B**), NRF1 (**C**), and TFAM (**D**) were measured by quantitative real-time PCR. The results from three biological replicates are shown as the mean ± SD. Significance was determined by one-way ANOVA with Šidák’s multiple-comparison test. ** *p* < 0.01. NS, not significant.

Mitochondrial content is intricately regulated through the coordinated interplay between mitochondrial biogenesis and mitophagy [23,24]. It has been shown that mitophagy subsequently induces a compensatory rise in mitochondrial biogenesis [25]. Consistently, levels of mitochondrial proteins SDHB and Cox2 initially decreased but gradually recovered (Figure 5A). Notably, co-treatment with compound C abolished this decrease and recovery (Figure 5A). To further verify whether berberine treatment stimulates mitochondrial biogenesis, we examined the expression levels of the major mitochondrial biogenesis regulators PGC-1α, NRF1, and TFAM through quantitative RT-PCR. The results indicated that the levels of PGC-1α, NRF1, and TFAM expression began to increase 48 h after berberine treatment (Figure 5B–D). These findings suggest that berberine treatment simultaneously induces mitochondrial biogenesis following mitophagy induction.

### 2.5. Berberine Ameliorates Mitochondrial Dysfunction in a PINK1 Knockout Model

**Figure 6:**
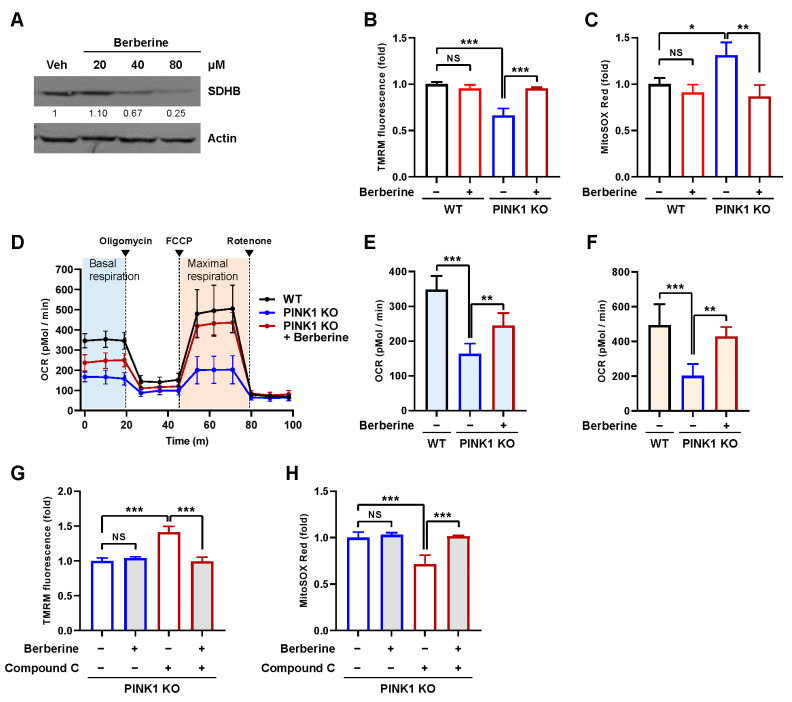
Effect of berberine on mitochondrial dysfunction in PINK1 KO MEFs. (**A**) PINK1 KO MEFs were treated with 20, 40, or 80 μM berberine for 24 h, and cell lysates were subjected to Western blot analysis using the indicated antibodies. Numbers below the corresponding blot represent densitometry values normalized to actin. (**B**–**F**) Wild-type (WT) MEFs and PINK1 KO MEFs were treated with berberine (80 μM) for 24 h and cultured for an additional 4 days. The mitochondrial membrane potential was assessed by TMRM staining (**B**), and mitochondrial ROS levels were determined by MitoSOX Red staining (**C**). The results from three biological replicates are shown as the mean ± SD. Mitochondrial respiration was analyzed by an XF-24 analyzer with five samples per group (**D**). Basal respiration (**E**) and maximal respiration (**F**) from mitochondrial respiration analyses are shown as the mean ± SD. (**G**,**H**) PINK1 KO MEFs were treated with berberine (80 μM) together with compound C (10 μM) for 24 h and cultured for an additional 4 days. The mitochondrial membrane potential (**G**) and mitochondrial ROS levels (**H**) were assessed. Significance was determined by one-way ANOVA with Šidák’s multiple-comparison test. * *p* < 0.05; ** *p* < 0.01; *** *p* < 0.001. NS, not significant.

Mitophagy has been shown to improve mitochondrial function in various mitochondrial dysfunction models [5,26,27]. To test whether berberine improves mitochondrial function, we treated PINK1 knockout (PNK1 KO) MEFs, a well-known model for mitochondrial dysfunction exhibiting decreased mitochondrial membrane potential, elevated mitochondrial ROS, and decreased mitochondrial respiration [28,29], with berberine. We treated PINK1 KO MEFs with different concentrations of berberine for 24 h, and mitophagy induction was analyzed by Western blotting for the mitochondrial protein SDHB. Similar to that in BEAS-2B cells, SDHB levels also began to decrease in PINK1 KO MEFs with 40 μM berberine treatment, and 80 μM treatment induced a clear reduction in SDHB levels (Figure 6A). Consistent with previous reports, PINK1 KO MEFs exhibited a decreased mitochondrial membrane potential of approximately 46% (Figure 6B). Notably, berberine treatment reversed the mitochondrial membrane potential to the level observed in wild-type MEFs (Figure 6B). Berberine treatment also reduced the elevated mitochondrial ROS level to the level observed in wild-type MEFs (Figure 6C). Additionally, the decrease in mitochondrial respiration was reversed by berberine treatment (Figure 6D). Both basal and maximal respiration increased by 50% and 113%, respectively (Figure 6E,F). These results suggest that berberine reversed mitochondrial dysfunction in PINK1 KO MEFs.

To verify whether berberine exerts its effect through AMPK-mediated mitophagy, we examined the effect of AMPK inhibitor compound C. We found that recovery of mitochondrial membrane potential and mitochondrial ROS upon berberine treatment was abolished by compound C (Figure 6G,H). These results further confirm that berberine reversed mitochondrial dysfunction in PINK1 KO MEFs through AMPK-dependent mitophagy induction.

## 3. Discussion

In this study, we observed that the natural alkaloid berberine promotes mitophagy activity in various human cell lines, including BEAS-2B human nontumorigenic lung epithelial cells, HeLa–Parkin cells, and A549 lung cancer cells. Unlike other well-known mitophagy inducers such as CCCP, berberine did not interfere with mitochondrial membrane potential. Additionally, berberine specifically induced a reduction in mitochondrial markers without causing the degradation of other subcellular organelles. These results suggest that berberine is a specific inducer of mitophagy without exerting toxicity on mitochondrial function.

Berberine has been studied for its potential effects on mitochondrial function, and several mechanisms have been proposed to explain its impact. Studies have suggested that berberine regulates the activity of the mitochondrial respiratory chain, which is responsible for generating ATP and stimulating mitochondrial biogenesis [30,31]. Berberine also possesses antioxidant properties, which can help protect mitochondria from oxidative stress [15,32]. In the present study, we showed that berberine improved mitochondrial function through the stimulation of mitophagy. Using the mt-Keima assay, a sensitive and reliable method for measuring mitophagy [16,17], we confirmed that berberine increases cellular mitophagy activity. Additionally, through further validation involving a decrease in mitochondrial protein and an increase in autophagosomes, we present the first direct evidence that berberine induced an increase in mitophagy activity.

Interestingly, previous studies have shown that berberine is involved in mitophagy regulation. An increase in the levels of mitophagy regulators, including LC3-II, PINK1, Parkin, and autophagosomes, has been observed upon berberine treatment in various experimental settings, for example, in a heart failure animal model, in influenza virus-infected macrophages, and in an acute kidney injury mouse model [33,34,35]. In addition, Wang et al. recently showed that berberine stimulates mitophagy through the inhibition of PINK1 promoter methylation [36]. These results suggest that berberine may promote mitophagy activity through various mechanisms in different cellular contexts. In this study, we identified that AMPK is responsible for berberine-induced mitophagy. Interestingly, many previous studies also observed AMPK activation upon berberine treatment and considered that this activation contributes to many of its physiological effects. For example, Li et al. showed that berberine protects retinal pigment epithelium (RPE) cells through the activation of AMPK [37]. Lee et al. also showed that the berberine-mediated activation of AMPK in adipocytes and myotubes is important for berberine to exert beneficial effects in insulin-resistant conditions [38]. Notably, in addition to its well-known function as a key regulator of cellular energy homeostasis, AMPK activation has been linked to the induction of mitophagy through various mechanisms [39].

AMPK can directly phosphorylate and activate ULK1, promoting the formation of autophagosomes and the subsequent engulfment of damaged mitochondria [40]. Additionally, AMPK can influence mitophagy by modulating the expression and activity of other key players in the process. For example, AMPK can phosphorylate and activate the transcription factor FOXO3a, which regulates the expression of genes involved in autophagy, including mitophagy-related genes [41]. AMPK stimulates mitophagy by increasing mitochondrial fission through the phosphorylation of MFF and Drp1 [42,43]. Moreover, AMPK can regulate the activity of the PINK1–Parkin pathway, another crucial mechanism in mitophagy [44]. In summary, AMPK activation is associated with the induction of mitophagy, influencing this process through the direct phosphorylation of key proteins involved in autophagy initiation and by regulating the expression and activity of downstream targets in the mitophagy pathway. Although Wang et al. previously showed that AMPKα2 rescues the impaired mitophagy through phosphorylation of PINK1 in a heart failure model [44], our results showed that berberine did not increase PINK1 levels. Berberine also reversed mitochondrial dysfunction in PINK1 KO MEFs, suggesting that AMPK regulates mitophagy through a different pathway. The precise mechanism by which berberine-induced AMPK activation regulates mitophagy induction needs to be investigated in future studies.

The results from numerous recent studies demonstrate that promoting mitophagy improves mitochondrial dysfunction and leads to therapeutic effects in various disease models, including heart diseases, metabolic disorders, and neurodegenerative diseases [5,26,27]. In our study, we demonstrated that berberine reversed mitochondrial dysfunction in PINK1 KO MEFs. Parkinson’s disease is often associated with dysfunction in the PINK1–Parkin mitophagy pathway [45,46,47]. Consequently, berberine treatment could be beneficial in a Parkinson’s disease model. However, it is important to note that although PINK1 KO MEFs provide valuable insight into cellular mechanisms related to PINK1 and certain aspects of Parkinson’s disease, their use alone might not fully capture the complexity of the disease. Thus, further investigation is needed to determine whether berberine administration indeed exerts therapeutic effects through mitophagy induction in a Parkinson’s disease animal model. Additionally, despite the traditional use of berberine across time, it is known to have low bioavailability, indicating that a significant portion of orally administered berberine may not be effectively absorbed into the bloodstream [48,49]. Strategies to enhance bioavailability of berberine, such as the development of novel formulations or coadministration with other compounds, should be explored. Further research is also necessary to ensure the maintenance of high blood concentrations of berberine and to investigate the mechanisms by which berberine traverses the blood–brain barrier for its therapeutic application in disease models. It is worth noting that the ability of berberine to traverse the blood–brain barrier can be improved through modifications in its dosage form.

Taken together, our results suggest that berberine exhibits mitophagy-inducing potential with low toxicity. Although the precise molecular mechanism by which berberine mediates mitophagy induction should be further investigated, our findings indicate that berberine has the potential to treat various diseases by improving mitochondrial dysfunction.

## 4. Materials and Methods

### 4.1. Cell Lines, Plasmids, and Treatments

BEAS-2B, A549, HeLa–Parkin, and MEF cells were maintained in Dulbecco’s modified Eagle medium containing 10% fetal bovine serum (FBS; JR Scientific Inc., Woodland, CA, USA). PINK1 KO MEFs were kindly provided by Dr. Jongkyeong Chung (Seoul National University, Seoul, Republic of Korea) [50]. Cell lines stably expressing mt-Keima or Keima were generated via infection with a lentivirus produced by using a pLVX-mtKeima lentiviral construct [23].

pLV-ER GFP (#80069), pLV-Golgi eGFP (#79809), and pmTurquoise2-Peroxi (#36203) were obtained from Addgene (Watertown, MA, USA). A mitochondrial YFP-expressing plasmid (pLESIP-mitoYFP) was generated by subcloning mitoYFP from pcDNA3-mitoYFP (provided by Dr. Gyesoon Yun, Ajou University, Suwon, Republic of Korea) into the pLESIP vector. CCCP (C2759), berberine (14050), bafilomycin A1 (B1793), and compound C (171260) were purchased from Sigma-Aldrich (St. Louis, MO, USA).

### 4.2. Measurement of Mitophagy and Autophagy Levels

To measure mitophagy activity through a flow cytometry-based assay, mt-Keima-expressing cells were treated with berberine, and mt-Keima fluorescence was examined using an LSR Fortessa flow cytometer (BD Biosciences, Franklin Lakes, NJ, USA) equipped with 405 nm and 561 nm lasers at the Neuroscience Translational Research Solution Center (Busan, Republic of Korea) as described previously [17]. The percentage of cells undergoing mitophagy (mitophagic cells (%)) was determined by gating cells exhibiting a high ratio of emission at 561 nm/405 nm excitation. To distinguish between high and low ratios of emission at 561 nm/405 nm excitation, we used untreated HeLa-mt-Keima cells exhibiting low mitophagy activity as the standard for a low ratio. The experiments were independently repeated three times and the results are presented as the mean ± SD.

To measure mitophagy levels in cells and tissue samples, mt-Keima fluorescence was detected using a Zeiss LSM 700 confocal microscope equipped with a C-Apochromat 40×/1.20 W Korr M27 lens at the Neuroscience Translational Research Solution Center (Busan, Republic of Korea). mt-Keima fluorescence was imaged using two sequential excitation lasers (458 nm and 561 nm) and a 595–700 nm emission bandwidth. Quantitation of mitophagy levels based on mt-Keima confocal images was performed using Zeiss Zen software (Zen 3.0 SR) as described previously [32,49]. We depicted the mt-Keima fluorescence signal from the 458 nm excitation wavelength in green and the signal from excitation by the 561 nm laser in red. The mitophagy level (mitophagy (%)) was defined as the number of pixels with a high red/green ratio divided by the total number of pixels. To quantify the mitophagy level in cells, the experiment was independently repeated three times, and at least five images per sample were analyzed in each experiment.

To measure autophagy levels in cells, Keima fluorescence was analyzed, and the level of autophagy (autophagy (%)) was determined in the same manner as the level of mitophagy described above. In all confocal microscopy analyses, all imaging parameters remained constant and only the gain level was adjusted to avoid the saturation of any pixel. All mitophagy and autophagy measurement results are presented as the mean ± SD.

### 4.3. Confocal Microscopy of Fluorescent Organelle-Specific Markers

To analyze the level of mitochondria and other organelles upon ATL001 treatment, cells expressing fluorescent markers for mitochondria (mitoYFP), the endoplasmic reticulum (ER: pLV-ER GFP), Golgi (pLV-Golgi eGFP), or peroxisome (pmTurquoise2-Peroxi) were examined using a Zeiss LSM 700 (Carl Zeiss, Jena, Germany) confocal microscope at the Neuroscience Translational Research Solution Center. To determine the fluorescence intensities of organelle-specific markers, at least five images per sample were analyzed. The experiment was independently repeated three times and the results are presented as the mean ± SD.

### 4.4. Western Blot Analysis

Cells were lysed in RIPA buffer and subjected to Western blot analysis as described previously [51]. Anti-Cox2 (ab198286), anti-Cox4 (ab16056), anti-SDHB (ab14714), anti-MFN2 (ab56889), anti-P4HB (ab137110), anti-PGC-1α (ab191838), anti-NRF1 (ab175932), and anti-TFAM (ab272885) antibodies were purchased from Abcam (Cambridge, UK). Anti-PDH (2784), anti-Acetyl-CoA Carboxylase (ACC) (3676), anti-phospho-Acetyl-CoA Carboxylase (pACC; Ser79) (3661), anti-AMPK (2532), and anti-phospho-AMPK (pAMPK; Thr172) (2531) antibodies were purchased from Cell Signaling Technology (Danvers, MA, USA). An anti-actin (SC-47778) antibody was purchased from Santa Cruz (Dallas, TX, USA) Band intensities were quantified using densitometry and ImageJ software (Image J 1.52k) (NIH, Bethesda, MD, USA).

### 4.5. Electron Microscopy

To analyze autophagosome formation, BEAS-2B cells treated with PDE701 were analyzed using an Apreo 2S LoVac scanning electron microscope (Thermo-Fisher Scientific, Waltham, MA, USA). Samples for electron microscopic analysis were prepared at the Neuroscience Translational Research Solution Center at Dong-A University. The cells and mouse tissues were then stained with 1% uranyl acetate, dehydrated with a graded ethanol series, and embedded in Epon. Ultrathin sections were cut on a microtome (Leica Microsystems, Wetzlar, Germany), placed on copper grids, and observed and photographed using an Apreo 2S LoVac scanning electron microscope. At least 12 cells per group were examined to determine the number of autophagosomes containing mitochondria in each cell.

### 4.6. Quantitative RT-PCR for Mitochondrial Biogenesis Regulators

For quantitative real-time PCR analysis, total RNA was isolated using an easy-BLUE™ Total RNA Extraction Kit (iNtRON Biotechnology, Seongnam, Republic of Korea) following the manufacturer’s instructions, and cDNA was synthesized using TOPscript™ RT DryMIX (Enzynomics, Daejeon, Republic of Korea). Quantitative real-time PCR was performed in triplicate in a SYBR Green PCR Master Mix (Enzynomics) using an ABI Prism 7500 Real-Time PCR System (Thermo Fisher Scientific, Waltham, MA, USA). Β-Actin was used as an internal control for all samples, with normalization of gene-specific mRNA levels to the β-actin RNA level. The level of each mRNA was determined using the 2^−ΔΔCΤ^-threshold cycle method.

The primers used were as follows: PGC-1α forward primer (5′-ATGTGTCGCCTTCTTGCTCT-3′) and PGC-1α reverse primer (5′-ATCTACTGCCTGGGGACCTT-3′); TFAM forward primer 5′-GTCCATAGGCACCGTATTGC-3′ and TFAM reverse primer 5′-CCCATGCTGGAAAAACACTT-3′; NRF1 forward primer, 5′-CAACAGGGAAGAAACGGAAA-3′ and NRF1 reverse primer 5′-GCACCACATTCTCCAAAGGT-3′; and Actin forward primer 5′-CATGTACGTTGCTATCCAGGC-3′ and Actin reverse primer 5′-CTCCTTAATGTCACGCACGAT-3′.

### 4.7. Analysis of Cell Death

BEAS-2B cells were treated with CCCP (10 μM) or berberine (80 μM) for 24 h and incubated for 3 days. Cell death was assessed using a FITC Annexin V Apoptosis Detection Kit 1 (BD Biosciences, Franklin Lakes, NJ, USA) according to the manufacturer’s protocol. The cells were analyzed using an Attune NxT cytometer (Thermo Fisher Scientific, Waltham, MA, USA), and Attune NxT Software 4.2.1672.1 (Thermo Fisher Scientific) was used to analyze the cell apoptosis rate. The experiment was independently repeated three times, and the results are presented as the mean ± SD.

### 4.8. Measurement of Mitochondrial Membrane Potential and Mitochondrial ROS

Mitochondrial membrane potential was measured using tetramethylrhodamine methyl ester (TMRM, 100 nM) (Invitrogen, Carlsbad, CA, USA) and mitochondrial ROS levels were measured using MitoSOX Red (5 μM) (Invitrogen, Carlsbad, CA, USA) as described previously [51]. The TMRM fluorescence intensity and MitoSOX Red fluorescence intensity were analyzed via flow cytometry using an LSR Fortessa cytometer.

### 4.9. Mitochondrial Respiration Analysis

Cellular respiration rates were measured in a 24-well plate using an XF24 flux analyzer (Seahorse Bioscience Inc., North Billerica, MA, USA) as described previously [51]. The oxygen consumption rate was measured under basal conditions followed by the sequential addition of oligomycin (0.5 μM), carbonyl cyanide *p*-trifluoromethoxyphenylhydrazone (FCCP, 1 μM), and rotenone (1 μM)/antimycin A (1 μM) to assess basal respiration, proton leakage, maximal respiration, nonmitochondrial respiration, and ATP production. The oxygen consumption parameters were normalized to the number of cells. The experiment was repeated five times and the results are presented as the mean ± SD.

## Data Availability

The data that support the findings of this study are available from the corresponding author, J.Y., upon reasonable request.
